# Natural Fungicolous Regulators of *Biscogniauxia* *destructiva* sp. nov. That Causes Beech Bark Tarcrust in Southern European (*Fagus sylvatica*) Forests

**DOI:** 10.3390/microorganisms8121999

**Published:** 2020-12-15

**Authors:** Vladimir Vujanovic, Seon Hwa Kim, Jelena Latinovic, Nedeljko Latinovic

**Affiliations:** 1 College of Agriculture and Bioresources, University of Saskatchewan, Saskatoon, SK S7N 5A8, Canada; sek049@mail.usask.ca; 2 Biotechnical Faculty, University of Montenegro, Mihaila Lalića 1, 81 000 Podgorica, Montenegro; jelenalat@ucg.ac.me (J.L.); nlatin@ac.me (N.L.)

**Keywords:** Tarcrust disease, beech forest, *Biscogniauxia*, fungicolous fungi, mycoparasites

## Abstract

Mycoparasites are a collection of fungicolous eukaryotic organisms that occur on and are antagonistic to a wide range of plant pathogenic fungi. To date, this fungal group has largely been neglected by biodiversity studies. However, this fungal group is of interest, as it may contain potential biocontrol agents of pathogenic fungi that cause beech Tarcrust disease (BTC), which has contributed to the devastation of European beech (*Fagus sylvatica*) forests. *Biscogniauxia nummularia* has been demonstrated to cause BTC. However, a trophic association between mycoparasites and pathogenic *Biscogniauxia* spp., has not been established. This study aimed to taxonomically identify and characterize *Biscogniauxia*, a fungus causing destructive BTC disease in European beech at Lovćen national park, Montenegro and to uncover the diversity of mycopathogens that are natural regulators of xylariaceous *Biscogniauxia* stroma formation, associated with beech decline. This finding is supported by distinctive phylogenetic and evolutionary characteristics, as well as unique morphological-microscopic fungal features indicating that *Biscogniauxia* from Montenegro, which is a major cause of BTC occurring in ancient beech forests at the edge of southern *Fagus sylvatica* distribution, may be described as a novel fungus specific to *Fagus.* Its evolutionary nuSSU–complete ITS–partial nuLSU rDNA phylogeny indicates its likely emergence by asexual fusion or introgressive hybridization between diverged *B. nummularia* and *B. anceps* species. The name *Biscogniauxia destructiva* is proposed for the novel fungus, as it is aggressive and highly destructive BTC disease.

## 1. Introduction

Mycoparasites are a typical trophic group of fungicolous eukaryotic organisms that occur on plant pathogenic fungal hosts across the kingdom fungi [1,2]. As potential biocontrol agents, mycoparasites merit particular scientific attention. Indeed, this fungal group is mostly neglected in biodiversity studies, and, thus, not characterized in detail [3]. Further, these beneficial organisms are naturally occurring antagonists that regulate destructive tree pathogen outbreaks in forestry systems. Due to the high ecological value associated with protecting ancient forests from climate change and decline [4], the mycobiome (myco = fungus) research focus is shifting towards the dynamics of beneficial fungal inhabitants of healthy and diseased trees, which, in turn, are indicative of ecological stability and integrity of forest ecosystems [5].

The decline of European beech (*Fagus sylvatica* L.) has been recorded throughout Europe, as well as Russia. This decline is linked mainly to beech bark disease (BBD), and, more recently, to the presence of *Biscogniauxia nummularia* (Bull.) Kunze under changing climatic conditions. *B. nummularia* is a serious primary beech bark pathogen belonging to the order Xylariales Nannf., which causes beech Tarcrust (BTC) disease [6]. This far, a trophic association between mycoparasites and pathogenic *Biscogniauxia* spp., occurring on the bark (trunk and branches) of *Fagus* spp., has not been established [6]. The objective of this study therefore was to decipher the diversity of mycoparasites, which are important as they provide natural protection against BTC.

**Figure 1:**
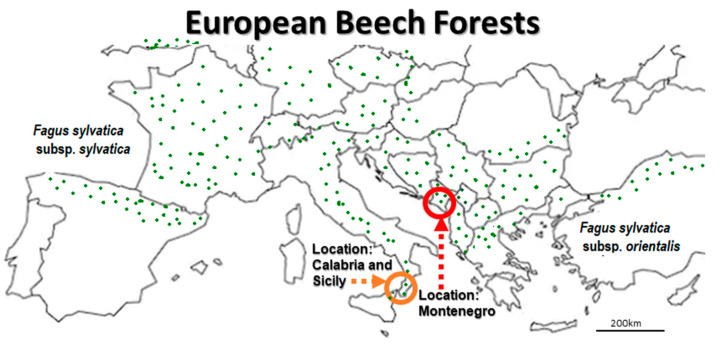
The European Beach (*F. sylvatica*) study location in Montenegro (red circle) is situated on the south-east Adriatic coast, and is in proximity to two locations on the south-west Adriatic coast, Calabria, and Sicily (orange cercle), Italy, where a highly virulent *Biscogniauxia*-Tarcrust (BTC) population has been detected [6]. Beech forests are regrouped in Western-, Central- and Southern European *F. sylvatica* subsp. *sylvatica* and Eastern European *F. sylvatica* subsp. *orientalis* taxa according to Peters [7].

Beech (*Fagus* L.) is a genus of deciduous broadleaf trees belonging the family Fagaceae. This genus is native to temperate Europe, Asia, and North America. A dozen or more beech species, including European beech (*F. sylvatica* L.) and American beech (*F. grandifolia* Ehrh.), are widespread [7] throughout the Northern hemisphere (Figure 1). Beech trees are economically, environmentally, and aesthetically important for national and regional economies, urban plantations, and recreational parks. However, beech forests are frequently affected by beech bark disease (BBD), which is an outcome of an insect (*Cryptococcus fagisuga* Lindinger)—fungi [*Nectria coccinea* (Pers.) Fr. and *N. ditissima* (Tul. and Tul.) Samuels and Rossman (=*N. galligena* Bres.) canker complex. Infected beech trees often die within 10 years, where the process of dying is usually accelerated by facultative pathogens such as Xylariales Nannf., and insects. Of the approximately 800 xylariaceous fungi described by Kirk et al. [8], Xylariaceae Tul. and C. Tul. (*Hypoxylon*, *Daldinia*, *Rosellinia*, and *Xylaria*) and Diatrypaceae Nitschke (*Diatrype*, *Diatrypetalla*, *Eutype* and *Eutypella*) dominate both European- and American beech species [5,9]. Approximately 10 fungal pathogens were registered as being associated with the bark disease complex per each study location, respectively, worldwide. Bark and wood-inhabiting fungi include anamorphs which occur as endophytic inhabitants in trees, shrubs, and herbs. Ascomycetous *Trichoderma* spp., have been screened for antagonistic ability against *Nectria* spp., in bark canker control, while *Beauveria* and *Lecanicillium* reportedly suppress *Cryptococcus* insects [10]. However, there is a lack of data regarding the biocontrol of xylariaceous fungi in beech bark, with particular reference to symptomatic BBD trees. The diversity of mycoparasitic fungi associated with *Biscogniauxia* Tarcrust (BTC) on European beech was compared with that of previously reported fungicolous species, which exhibit mycoparasitism on Xylariales hosts (Table 1). Among all listed fungi in Table 1, *Cosmospora* (Nectriaceae) species were reportedly the most widespread mycoparasitic or fungicolous fungi occurring on carbonized perithecia of Xylariales members [11]. Vujanovic et al., (2003) discovered *Cosmospora episphaeria* specific hyperparasitism against *Hypoxylon* spp. on colonized *F. grandifolia* bark in North America [12]. However, information regarding the diversity and structure of mycoparasitic or antagonistic fungi on *Biscogniauxia* spp., such as *B. nummularia*, is lacking [13].

The primary objective of the current study was to taxonomically identify and characterize *Biscogniauxia* in European beech at Lovćen NP, Montenegro. The study site consisted of a natural forest located at the extreme southern border of *F. sylvatica* habitat in Europe. A subsidiary objective was to uncover the diversity of mycopathogens that are the natural regulators of xylariaceous *Biscogniauxia* stroma formation, associated with beech decline. The results of this study are discussed in the light of increased interest in biological control agents as an environmentally friendly solution to the destruction of natural beach forest ecosystems by a combination of BBD and BTC diseases.

## 2. Materials and Methods

### 2.1. Location and Sampling

Lovćen, a national park in south-eastern Europe (Figure 1), is located in a mountainous region on the Adriatic coast of south-west Montenegro [Google Maps. “Lovćen NP, Montenegro”. Accessed 15 August 2019. https://www.google.com/maps/place/Lov%C4%87en/@42.3994425,18.7759734,12z/data=!4m5!3m4!1s0x134dcd307d6d2e33:0x37926cb0848a8933!8m2!3d42.3994444!4d18.8188889!5m1!1e4]. It is an attractive, mountainous touristic region situated between the bay of Kotor (UNESCO World Heritage Site), Budva (a preserved medieval town), and Cetinje City (the former royal capital). *Fagetum montenegrinum montanum* Bleč is the dominant forest ecosystem, where continental climatic conditions (750–1300 m altitude) are strongly influenced by the Mediterranean precipitation regime (~2000 mm) with high rainfall in spring and autumn, and a relatively dry summer [9]. The BBD-BTC stands on calcareous rocks and dolomite are of poor quality, while unhealthy European beech trees are mainly colonized by BBD causing *Neonectria/Nectria* spp., ascomycetous pathogens, BTC causing *Biscogniauxia* sp. (=*Hypoxylon* sp.), xylariaceous pathogens and wood-decaying *Ustulina*, *Fomes*, *Melanopus*, *Pleurotus*, *Chrondostereum* and *Ganoderma* associated with *Agrilus* insects (fam. Buprestidae) [20].

**Figure 2:**
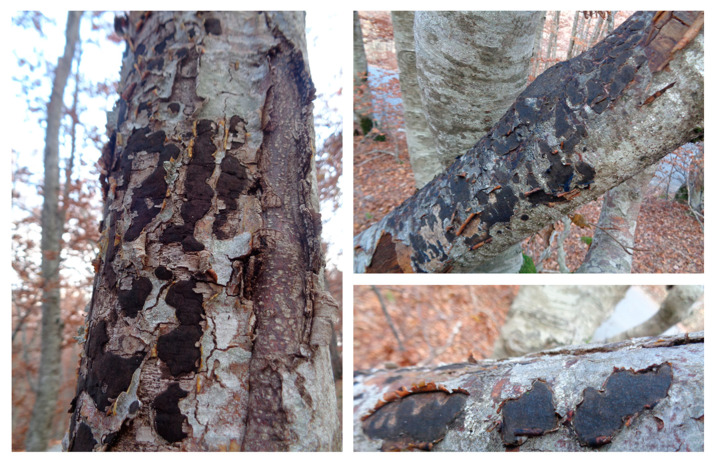
Beech tree dominated forest containing trees exhibiting beech bark disease (BBD), which is associated with carbonaceous black-stroma indicative of Tarcrust symptoms (‘dark chromatism’), caused by destructive *Biscogniauxia* sp. nov. pathogen, on both dying trees and dead logs.

Sampling was conducted within a 4 km^2^ assessment zone in an ancient European beech forest area at Ivanova Korita, Montenegro (Coordinates: 42°23′57″ N 18°49′06″ E/42.3991° N 18.8184 18° E). Bark samples displaying Tarcrust disease symptoms and stromata were obtained from the *F. sylvatica* forest in 2018 and 2019. Sampling consisted of 5 carbonized stroma samples per tree × 5 replicates, bearing perithecia of xylariaceaus *Biscogniauxia*. Samples were drawn from symptomatic Tarcrust stroma, in the form of a pronounced carbonaceous black plaque on beech bark, over a gradient of BTC symptoms and associated tree health statuses as follows: (i) diseased, (ii) dying, and (iii) dead (Figure 2).

### 2.2. DNA Extraction, Sequencing Methods and Microscopy

Bark samples with *Biscogniauxia* carbonaceous stromata symptomatic of Tarcrust were collected from beech trunks at ~1.5 m height, surface sterilized (alcohol—90%) and processed in a Biosafety 2 level laboratory using sterile Graham-Field^TM^ stainless-steel blades (ThermoFisher Scientific, Saint-Laurent, QC, Canada). Morphological categorization of fungal sexual (ascomata and ascospores) and asexual (mycelia and conidia) structures were performed using standard laboratory procedures, using a Carl Zeiss Axioskop2 with a Carl Zeiss AxioCamICc1 (Göttingen, Germany) camera [21].

Genomic DNA from surface sterilized xylariaceous stromata samples (10 s alcohol—75%, 1 min sodium hypochlorite—1.5%, and rinsed thrice in SDW) were extracted using an UltraClean Microbial Kit (Qiagen Inc., Mississauga, ON, Canada), according to the manufacturer’s instructions. Fungal diversity was profiled via Universal ITS1-ITS4 primers [22], which amplify a partial nuSSU–complete ITS–partial nuLSU rDNA region [23], as described in Jaklitsch et al. [24]. When amplification was unsuccessful for certain fungicolous or mycoparasitic species, *nuLSU* was amplified using primer sets NS1/NS6 and LS1/LR5 [21]. Thus, LSU rDNA sequences were used as an alternative for assessing the phylogenetic importance of distinctive morphological features observed in fungal samples via microscopy. All obtained amplicons were amplified and sequenced by the NRC (National Research Council), Canada. These obtained sequences were analyzed against the GenBank database (NCBI) to identify primary BTC-Tarcrust pathogens and associated fungicolous and mycoparasitic, endophytic and facultative fungicolous saprotrophic fungal genotypes.

In order to reveal the phylogenetic classification of the host fungus within Xylariales, we investigated the relationships and delimitations within *Biscogniauxia* spp., via phylogenetic analyses. Fungal evolutionary history was analyzed using the minimum-evolution method [25], while evolutionary distances were computed using the maximum composite likelihood method [26]. The minimum evolution (ME) tree was searched using the Close-Neighbor-Interchange (CNI) algorithm [27], while the neighbor-joining algorithm [28] was used to generate the phylogenetic tree. Following the removal of all ambiguous positions, evolutionary analyses were conducted in MEGA X -version 10.0.5 [29]. Nucleotide sequences were deposited in NCBI/EMBL GenBank, under the accession numbers that are provided in the text and figures containing phylogenetic trees.

## 3. Results

### 3.1. Biscogniauxia sp. Taxonomy and BTC Identity

**Figure 3:**
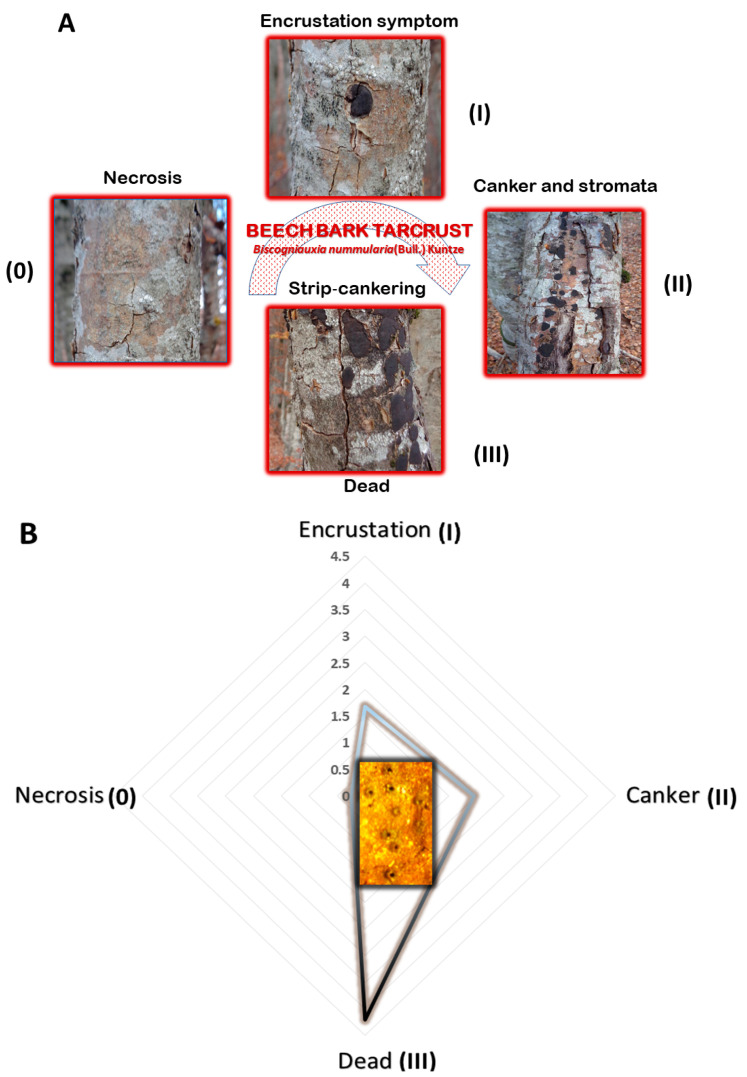
Disease curve (signs and symptoms) depicting important changes in beech bark Tarcrust symptomatology, increased tree decline, and death. (**A**) progress of bark disease symptoms without (0) and with developed stroma (I, II, and, III); and (**B**) increased stroma size (cm^2^) from the initial (0) to advanced disease stages (Note: stroma shows papillate ostioles).

A taxon-wide nuclear ribosomal DNA inventory, supported by microscopy, revealed a remarkable diversity of 25 fungal genera of *Ascomycota* and *Basidiomycota*. The etiology of *F. sylvatica* decline in Montenegro is directly related to combined BBD and, aggressively spreading *Biscogniauxia* sp. Beech Tarcrust (BTC) outbreaks. In particular, the Lovćen NP forest area is under an increasing BCT progress curve dominated by *Biscogniauxia* sp. symptomatology (Figure 2 and Figure 3A). In most European beech forests, *B. nummularia* (Bull.) Kuntze (1891) (basionym: *Hypoxylon nummularium* Bull., Herb. France (Paris)) is considered to be the predominant cause of Tarcrust disease in *F. sylvatica*. According to the systematics of Pyrenomycetes and the Dichotomous key to European *Biscogniauxia* taxa [30], the morphology of *B. nummularia* (Bull.) Kuntze., is distinct from that of all other known *Biscogniauxia* spp. Among the major distinctive characteristics listed are applanate stromata with black stromatal surfaces, with slightly papillate ostioles and blackish brown ellipsoid ascospores, which are indicative of specificity for *Fagus*. These have been recorded in all parts of Europe and Russia, where its host is distributed. Rogers, et al. [31], described similar disease symptomatology for *Biscogniauxia anceps* (Sacc.) J.D. Rogers, Y.M. Ju & Cand. which is the only non-specific Tarcrust disease on trees and shrubs in Europe. It is frequently found on the bark of *Corylus avellana* and to a lesser extent on *Acer, Crataegus, Fraxinus*, and *Laurus*. This is a rare species, of which *Fagus* is not a host. It is largely restricted to areas under oceanic and Mediterranean influence such as Italy, France, Spain, and the UK. Interestingly, *B. anceps* is the only European species of *Biscogniauxia* known to have bicellular (two-celled) ascospores with typical dark colored and hyaline cells. This feature is considered neotenic [31], which indicates that sexual maturity and reproduction of the organism is attained by the retention of juvenile characteristics, represented by the presence of hyaline germinable ascospores. Despite such morphological distinctions between *B. nummularia* and *B. anceps*, a recent rDNA phylogenetic analysiss suggested that these two species belong to the same evolutionary clade [32] and, therefore, the only two fungal taxa to be retained within genus *Biscogniauxia*, thus far.

### 3.2. Unique Biscogniauxia-BTC Taxon on Fagus in Montenegro

**Figure 4:**
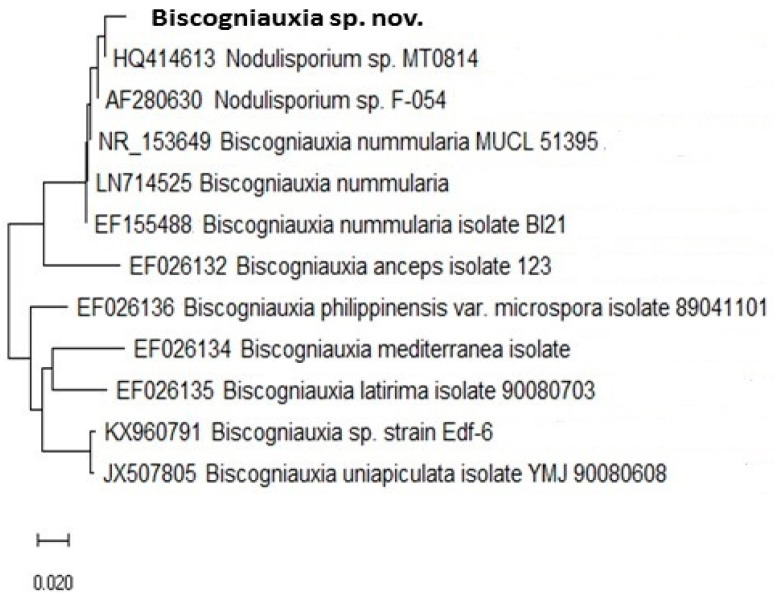
Phylogenetic tree of ***Biscogniauxia*** taxa, including ***B. destructiva*** sp. nov. (GenBank acc. no. MT804371) from Tarcrust stroma samples, constructed using nuSSU–complete ITS–partial nuLSU rDNA sequences. The sequences used showed >95% similarity with the sequences deposited in the NCBI database (www.ncbi.nih.gov). Evolutionary history was inferred using Mega X software. The optimal tree with a sum of branch length = 0.58037206 is shown. The bar represents number of expected substitutions accumulated per site.

**Figure 5:**
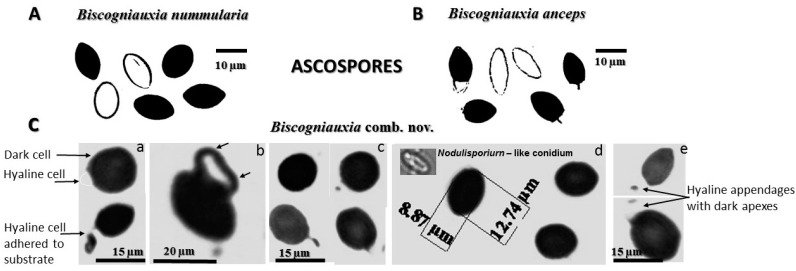
*Biscogniauxia destructiva* exhibits ascospore characteristics that are distinctive from its two sister species: (**A**) *B. nummularia*, with unicellular-dark and unicellular-hyaline ascospores without appendages (Herbarium: JF-99047); (**B**) *B. anceps* shows a bicellular ascospore with superior dark cell and inferior hyaline cell, which bears a stick-like appendage, and unicellular-hyaline ascospore without an appendage (Herbarium: JF-02199); and (**C**) *B. destructiva*, which is characterized by a combination of unicellular-dark and bicellular-dark, hyaline ascospores. C-a (above): dark cell (with germ slit) and hyaline cell (without germ slit) adherent to the substrate/host/vector tissue; C-a (below): mucilaginous-sticky appendages attached to the substrate; C-b: germinated dark-walled cell showing two germ tube protruding vesicles with two apexes (arrows); C-c: typical ascospores with appendages; C-d: *Nodulisporium*-like anamorphic spore (conidium); C-e: hyaline trade-like appendages with a dark apex.

The current study discovered a very aggressive *Biscogniauxia* population which induces destructive Tarcrust formation on *F. sylvatica* in Montenegro (Figure 3). Analyzed *Biscogniauxia* samples showed unique taxonomic features (Figure 4 and Figure 5). Its fungal rDNA phylogeny (Figure 4), combined with morphological characteristics, as well as microscopic (teleomorph and anamorph) features (Figure 5), characterize it as a distinct fungal taxon (Table 2), identified as *Biscogniauxia* sp. nov. (GenBank acc. numbers: MT804371) due to its differences from other *Biscogniauxia* spp. The findings of this study indicate that recent monophyletic evolution of *B. nummularia* has resulted in advanced fungal speciation. The phylogenetic tree (Figure 4), based on nuSSU–complete ITS–partial nuLSU sequences, shows the interesting evolutionary topology of this novel *Biscogniauxia* taxon, which appears to be a distinct rDNA variant of its sister *B. nummularia* (97.23% similarity to MUCL 51395 type material; GenBank acc. number: NR_153694), found in France and Germany, as well as of *B. anceps* (90.29% similarity to GenBank acc. number EF026132) found in France.

*Biscogniauxia* from Montenegro may be described as a novel fungus specific to *Fagus*. It is a major cause of BTC occurring in ancient beech forests at the edge of southern *F. sylvatica* distribution. These findings are supported by distinctive phylogenetic and evolutionary characteristics (Figure 4), as well as unique morphological-microscopic features (Table 2), and, thus, the name *Biscogniauxia destructiva* is proposed, as follows.

*Biscogniauxia destructiva* Vujan.

Anamorph: *Nodulisporium*

Host: *Fagus sylvatica* L.

Location: Lovćen NP (Montenegro)

Sample: Fungal stroma; Collected: 02-07-2018

GenBank acc. number: MT804371

The etymology of *destructiva* demonstrates that it is derived from the Latin word ‘*destruere*’, which refers to causing damage, being destructive or devastating (to beech forest habitat in this case). This distinct *Biscogniauxia* has an anamorphic *Numularia* stage as defined by Ju and Rogers [33]. *Biscogniauxia* produce conidia holoblastically in sympodial sequence [34]. Its taxonomic identity was also confirmed in this study by both phylogenetic (Figure 4) and microscopic spores features (Table 2 and Figure 5).

### 3.3. Endophytic Fungicolous Fungi, Mycoparasites and Hyperparasites

**Figure 6:**
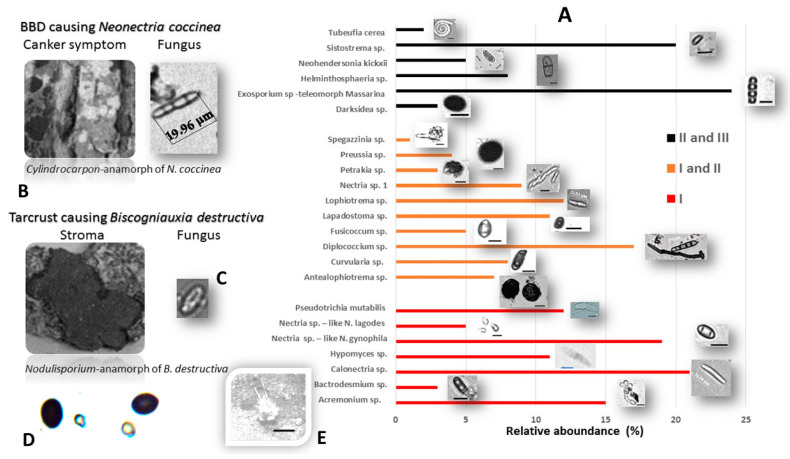
(**A**) Fungicolous and mycoparasitic fungi discovered on Tarcrust stromata of *Biscogniauxia destructiva* hosts in *F. sylvatica* forests (Lovćen NP, Montenegro); (**B**) *Cylindrocarpon*-anamorph of *Nectria coccinea*; (**C**) *Nodulisporium*-anamorph of *B. destructive*; (**D**) *Calcarisporium xylariicola* (Hypocreales) conidia (<5.5 μm size according to Sun et al. [36] were usually mixed with dark *B. destructiva* ascospores (~10 μm size) on the surface of xylariaceous stromata (left-down); (**E**) *Phyllactinia* sp. (Erysiphales) powdery mildew cleistothecium (center-down) an obligate fungicolous taxon was also found on the stroma’s surface.(scale bar: 10 μm).

**Figure 7:**
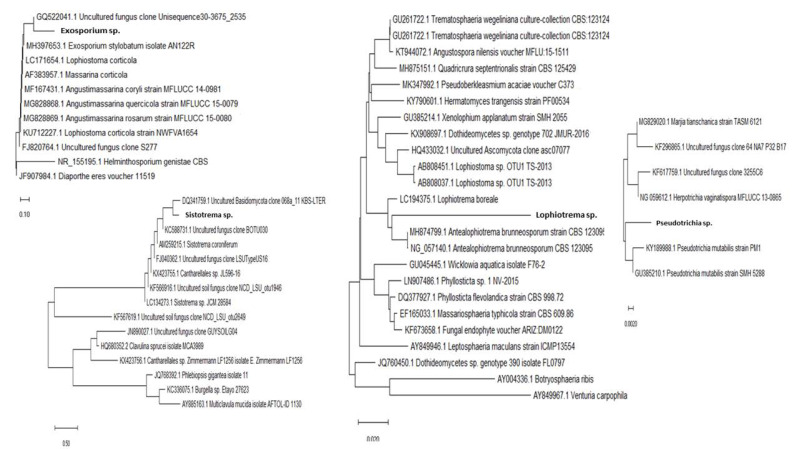
Phylogenetic position of four interesting fungicolous or mycoparasitic fungi discovered on pathogenic *Biscogniauxia destructiva* sp. nov.-host fungus stromata (GenBank. Acc. no. MT804371) in *F. sylvatica* forests (Lovćen NP, Montenegro) based on ITS (*Exosporium* sp., GenBank acc. no. MT799884 and *Pseudotrichia* sp. MT807909) and LSU (*Sistotrema* sp. GenBank acc. no. MT804547 and *Lophiotrema* sp. MT804550) sequences. The rDNA sequences used show >95% similarity with sequences deposited in the NCBI database (www.ncbi.nih.gov). Evolutionary history was inferred using Mega X software. The optimal tree with a branch sum length >0.75065795 is shown. Bars represent number of expected substitutions accumulated per site.

BLASTn analysis of nuclear ribosomal ITS and LSU sequences combined with microscopic observations were used to identify the endophytic fungicolous/mycoparasitic inhabitants of Tarcrust stroma (Figure 6 and Figure 7).

Most fungicolous species discovered during the course of this study have not been previously reported on beech (*Fagus*). Some of these are neither described nor reported as fungicolous fungi on Xylariales in the Web of Science database (1990–2020). Twenty-three fungicolous taxa were found on *Biscogniauxia* sp. nov. (anamorph *Nodulisporium*)-Tarcrust stromata samples collected from BBD beech forest trees, including the rarely present *Neonectria coccinea* (anamorph: *Cylindrocladium* according to Chaverri et al. [35]), are shown (Figure 6). Microscopic images representing each detected fungicolous taxon are also provided. These distinctive morphological data (Figure 6), when combined with rDNA sequences, blasted against GeneBank database and phylogenetically analyzed (Figure 7), confirmed a diversified taxonomic appurtenance of fungicolous fungi on *B. destructiva* sp. nov.-BTC, in Montenegro. Unique genomic rDNA (Figure 7.) enabled detection of *Exosporium* sp. (GenBank acc. no. MT799884, ITS), *Pseudotrichia* sp. (MT807909, ITS), *Sistotrema* sp. (GenBank acc. no. MT80454 LSU) and *Lophiotrema* sp. (MT804550, LSU) sequences. Some of these, as well as other fungicolous taxa, such as *Darksidea* sp. (GenBank acc. no. MT804548 LSU), *Petrakia* sp. (MT804549, LSU) and *Sistotrema* sp. (MT804547, LSU), may represent undescribed species.

The distribution and abundance of fungicolous fungi on *Biscogniauxia* stromata (Figure 6) define the potential of resident fungal diversity and the capacity of the mycoparasitism to naturally control BTC in beech forests. The significance of each described taxon may be measured by its occurrence in relation to stroma size dynamics across the development and progress of BTC stroma through stages I, II, and III, via the disease curve (Figure 3). The fungicolous community was represented by 30% taxa specific to stroma developmental-stage I, compared to 44% taxa specific to stages I and II and 26% taxa associated with stages II and III. At stroma initiation-stage I, *Calonectria* sp., *Nectria* spp., *Acremonium* sp., *Pseudotrichia* sp. and *Hypomyces* were predominant, followed by *Bactrodesmium* sp. During stages I-II, *Diplococcium* sp., *Lophiotrema* sp., *Antealophiotrema* sp., and *Nectria* sp.1 predominated, followed by the subdominant *Curvularia* sp., *Preussia* sp. and *Fusicoccum* sp., and the rarely occurring *Petrakia* and *Spegazzinia* spp. At stages II and III, *Exosporium* sp. and *Sistostrima* sp. predominated, followed by *Helmithospheria* sp. and *Neohendersonia* sp., while *Darksidea* sp. and *Tubeufia* sp. were only recorded relatively rarely.

## 4. Discussion

### 4.1. Tarcrust Identity, Pathogenicity and Symptomatology

Although the current concepts regarding *Biscognauxia* were defined by Pouzar [37] and revised by Ju et al. [33], a recent phylogenetic rDNA analyses suggests that *B. nummularia* belongs to a clade that is separate from other *Biscogniauxia* spp. This substantiates the data of Wendt et al. [38], which phylogenetically placed *B. nummularia* (France, type species) outside other known *Biscogniauxia* species. U’Ren et al. [32], have classified *B. anceps* as a species that is phylogenetically close to *B. nummularia*, indicating their common affinity with Xylariaceae, rather than with Graphostromataceae, as suggested by previous taxonomic studies. Although not closely related to *B. nummularia* and *B. anceps*, it seems important to indicate that *B. mediteranneum* (=*H. mediterraneum*) population, a cause of Tarcust disease on *Quercus* (Oak), in Europe, demonstrated a certain level of genomic plasticity and speciation. Consequently, this species has been reclassified based on morphology into two distinct taxa, namely *B. mediterraneum* var. *mediterraneum* and *B. mediterraneum* var. *microspora* [39,40]. The level of speciation in the rest of *Biscognauxiataxa* taxa is still unknown.

From a pathological standpoint, the *Biscogniauxia destructiva* population in Montenegro represents a new fungal taxon that occupies the ancient mountainous *F. sylvatica* forests distributed throughout the southern coast of the Adriatic Sea basin. This experimental site was situated in an area of Montenegro, which is immediately opposite neighboring Calabria and Sicily (Figure 1), where Granata and Sidoti [6] discovered the highest degrees of pathogenicity among *B. nummularia* geographical populations across Italy. Artificial fungal inoculation using *B. nummularia* isolates from the extreme southern habitats (Calabria and Sicily) of *F. sylvatica* has resulted in significantly higher fungal pathogenicity and larger cankers on *F. sylvatica* bark, leading to more wood darkening. This is corroborated by the devastating effects of *Biscogniauxia* sp. nov. on increasingly declining European beech forests in Montenegro [9,41]. High pathogenicity of *Biscogniauxia* at the extreme southern end of *F. sylvatica* distribution on both coasts of the Adriatic Sea suggests that further studies are needed to assert whether *Biscogniauxia* in Italy belongs to the same fungal population discovered in Montenegro.

Important changes in Tarcrust symptomatology and the increasing decline in trees (Figure 2) concur with data presented in Figure 3A progress in bark disease symptoms resulting in tree deaths and, Figure 3B) increases in stroma size (cm^2^), corresponding to the level of beech forest destruction. The southern host distribution area appears to be conducive to predomination by ‘strip canker’ symptoms (Figure 3A), which coincided with a year of massive beech fructification followed by water stress [9,41]. A latent infection of *Biscogniauxia* in *F. sylvatica* was proposed as a possible bioindicator of the health condition of beech trees [42]. In this study, *Nodulisporium* was found to be an anamorph of *Biscogniauxia* sp. nov. Its sister, *B. nummularia,* also produces anamorphic *Nodulisporium*, which mostly appears as a saprotroph on dead material, but may exist asymptomatically as an endophyte in healthy *Fagus* bark and leaf tissues (U’Ren et al. [32]). Its endophytic stage has been also reported in the tissues of co-dominant *Fraxinus* [43], as well as *Carex* grasses [44] in the understory of beech forests. Similar to *B. mediterranea*, this fungus spreads rapidly following a period of drought, and a large number of black stromata harboring perithecia erupt simultaneously from dead bark [45]. These are important ecological traits that may be attributed to *Biscogniauxia* sp. nov as well, which better explains its behavioral etiology. Additionally, insects, such as *Agrilus viridis* [20], which are frequently found on *F. sylvatica* trees, may act as possible natural vectors. Moreover, a strong association has been suggested between *B. nummularia*-Tarcrust fungal damage, jewel beetles (*A. viridis*), and beech bark beetles (*Taphrorychus bicolor*), as indicated by larval galleries and exit holes found in upper trunk portions, showing fungal infection [46]. The presence of the ascospore appendage (Figure 5C-e), may also indicate that *Biscogniauxia* sp. nov., attempts to maximize the dispersion of ascospores as spring and fall precipitation picks up. Beech tree architecture is characterized by a centripetal water retention system allowing descendent transportation of ascospores from crown to trunk (bark), soil, or root. Furthermore, the appendages of this fungus display a mucilaginous apex that is able to attach to the substrate of the host bark (Figure 5C-a) and possibly insect vectors. Ascospores that attach to insect vectors via the sticky apex of the appendage may enable the disease to spread widely and in new regions, particularly during increased insect activity.

### 4.2. Biscogniauxia sp. nov. Evolutionary Affinity

Overall, *Biscogniauxia* sp. nov. exerts a unique evolutionary affinity and specificity for *Fagus* host. Further, its rDNA sequence shows similarity to a distinct sister, *B. nummularia*, rather than to *B. anceps* species. While the presence of one-celled ascospores resembles that of *B. nummularia*, the production of two-celled ascospores with appendages places this species closer to *B. anceps*. However, the absence of one-celled hyaline ascospore separates *Biscogniauxia* sp. nov. from the two species stated above, indicating that continuous speciation of this fugal population is taking place in southern Europe. We assume that this particular combination of spore features may arise from more than one mutation or via interspecies hybridization. Indeed, the SSU-ITS-LSU phylogeny suggests a possibility of the emergence of *Biscogniauxia* sp. nov. through *Nodulisporium*-asexual introgressive hybridization between diverged *B. anceps* and *B. nummularia* species. Stukenbrock [47] have addressed this phenomenon in relation to asexual fusion in fungi and the role of hybridization in evolution and emergence of new fungal plant pathogens. Interestingly, Rogers et al., (1996) have pointed out that *B. anceps* is taxonomically unsettled, due to its neotenic situation, exemplified by the reversion of ascospores to a more juvenile or ancestral condition [31]. It appears that *Biscogniauxia* sp. nov. has reversed this particular ascospore characteristic, retaining only the black cells. Hence, this novel taxon may have re-implemented its typical affinity to xylariaceous fungi within fam. Xylariaceae, instead of fam. Graphostromataceae. In this fungus, the two-celled, colored ascospore with a smaller and slightly less colored, inferior cell that bears the appendage might be the result of cross hybridization between *B. nummularia* and *B. anceps*. The latter may be explained by the fact that a shift in the fungal genome may have resulted in evolutionarily adaptive vegetative growth in the form of a series of asexual reproduction steps resulting in *Nodulisporim* clonal offspring, while taking a relatively short evolutionary time to establish fully sexual *Biscogniauxia* reproduction. The ancestral characteristic of the appendage, eventually inherited from *B. anceps*, gained a mucilaginous apex that further improved cellular functionally, allowing the ascospore to float in water and attach itself to a substrate such as an insect body. For other eukaryotic fungi [48], this type of appendage may be evolutionarily advantageous in adapting to a terrestrial fungal lifestyle, as it allows effective transmission of ascospores via water and insect vectors, thus, enhancing disease spread and pathogenicity.

Further, multiple taxonomic and BTC pathogenicity traits point to the possibility that *B. nummularia* coexists with *Biscogniauxia* sp. nov. populations across European beech forest habitats. However, several distinctive morphological characteristics of *Biscogniauxia* sp. nov., reinforced by evolutionary relationships with both *B. nummularia* and *B. anceps* and its southern European localization on *Fagus*, suggest its geographic delimitation from central and northern European *B. nummularia* populations. In addition, the geographic proximity of *F. sylvatica* L. (European beech) and *Fagus orientalis* (Oriental beech) Lipsky throughout the Balkan Mountains of southern Europe, between the Adriatic and Black Seas, make both of these suitable hosts for *Biscogniauxia* pathogens. *B. nummularia* was discovered some time ago in a *F. orientalis* wood (LE 126997 and 127010, TYPES; Krasnodar Terr., Klutschewaja, Prov. Kuban, Caucasi bor., VI.1911), in the Black Sea basin of the Kuban region (= *Nummularia bulliardi* Tul. & C. Tul. var. *minor* Rehm, Transzchel, and Serebrianikow, Mycotheca Rossica Sive Fungorum Rossiae Et Regionum Confinium Asiae Specimina Exsiccata, fasc. 6 & 7, no. 277. 1912.), which posed a further dilemma regarding the relationship between the *Biscogniauxia* populations throughout south-western south-eastern European habitats across the beech forest regions of Italian and Balkan Peninsulas. Complexities associated with the origin of *Biscogniauxia* sp. nov. compared to *B. nummularia* and *B. anceps* are linked to the genotypes of the two primary *Fagus* hosts. These two *Fagus* species have integrated via hybridization into typical hybrids named *Fagus* × *taurica*. The habitat of this hybrid is restricted to mountain forests, at an altitude of 500–2100 m [49], similar to that of Montenegro beech forests. However, there is no data confirming the presence of *Fagus* × *taurica* in Montenegro beech forests. Future tree breeding programs may assist in addressing a *Fagus*’s host genotype issue in Montenegro and improving beech trees resistance against *B. nummularia* and *B.* sp. nov. populations, as these two BTC pathogens are specific to *Fagus* in Europe.

### 4.3. Fungicolous Fungi

Interestingly, pathogenic *Biscogniauxia* populations in both Montenegro (this study) and neighboring Italy [6] have induced pronounced subcortical darkening, which tends to spread upwards, on the trunks of beech. This indicates a systematic spread of the fungus and possible antagonism or biocontrol effect exerted on *Biscogniauxia* by surrounding fungal inhabitants. Fine black ‘demarcation’ lines are usually formed between pathogenic and antagonistic fungi [50], resulting in unpigmented areas of wood. These demarcation zones are usually associated with melanin deposition, which is usually enhanced to limit access to fungal competitors, and as results of limited water availability [51].

These findings corroborate those of previous studies encompassing the fungicolous genera within Ascomycota and rarely, Basidiomycota [52]. To the best of our knowledge, this is the first report on the presence of *Sistostrema* sp. among the fungicolous fungi pertaining to Basidiomycota. Although most listed fungicolous Ascomycota have been reported on different xylariaceous hosts, only *Pseudotrichia mutabilis* [17] has been reported as a mycoparasite on old stroma containing *Biscogniauxia marginata* (Fr.) Pouzar in eastern Europe (Lithuania). None of the other 22 taxa, or 96% of this study inventory, have been reported as being associated with *Biscogniauxia* spp. stromata. However, most of these fungicolous taxa have been exemplified as being fungicolous on various fungi, including other xylariaceous taxa (Table 1). There is a spectrum of mycoparasites in highly polyphyletic fungi, such as *Acremonium* spp., including *A.* *crotocinigenum*, *A.* *bactrocephalum*, *A. egyptiacum* or *A. kiliense*, and A. strictum, among others [36,53,54,55]. Other mycoparasites/hyperparasites are more host specific, such as *Calonectria,* which includes the obligate fungicolous *C. gymnosporangii* [56] and *Calcarisporium* sp., which includes the obligate fungicolous *C. xylariicola* on carbonaceous stroma containing *Xylaria* sp. [36]. Endophytic nature has been recognized in *Darksidea* spp., such as *D. epsilon,* which is a dark septate endophyte (DSE) found in stressed plants under semiarid conditions [57], similar to that of beech forests in Montenegro. *Fusicoccum* sp., a possible anamorph of *Botryosphaeria*, may also act as a multi-host endophytic fungus [58]. Furthermore, *Diplococcium* sp., such as *D. heterosporum* [3,59], and *Exosporium* sp., such as *E. stilbaceum* and *E. ampullaceum* co-occur with various fungicolous taxa [3,60]. Although *Exosporium* has been recently re-examined and re-described by Guatimosim et al. [61], the taxa in this genus are frequently confounded with those of *Cercospora* and *Helminthosporium* [62], which are also known as fungicolous taxa. Moreover, *Curvularia* sp. may be close to one of the multiple fungicolous *C. pallescens, C. lunata, C. leonenis, C. intermedia, C. cymbopogonis,* and *C. andropogonis* taxa [1,63]. *Hypomyces* sp., is an additional obligate fungicolous taxon [64] similar to *H. papyraceus* on *Ustulina deusta* carbonaceous stroma [17]. *Nectria* also contains several mycogenous taxa such as *N. episphaeria, N. pseudepisphaeria* and *N. triqua* [13,15,17]. However, endophytic *Nectria* spp. described here possess unusual ascospores similar to those of *Nectria lagodes* and *N. gynophila* species [65], indicating that, eventually, mosses/bryophites growing on the edge of Tarcrust stroma may act as primary/secondary host species. Among the fungicolous pyrenomycetes found, there were *Helminthosphaeria* spp., such as *H. corticiorum* and *H. fungicolous* [59]. Various other fungicolous Insertae Sedis *Petrakia* sp., such as *P. irregularis* [17] pleosporaceous *Pseudotrichia* sp., such as *Pseudotrichia mutabilis* [17] and pleosporomycetous *Tubeufia* spp., such as *T. cerea* and *T.* *brevispina* [13,17,66] were found. Some interesting fungicolous *Tubeufia*, such as *T. heterodermiae*, also showed a lichenicolous nature, appearing on stressed or dying trees [67]. Although *Cosmospora episphaeria* has been previously reported on carbonaceous stroma of Xylariales [11,12], it appears that *B. destructiva* sp. nov. is not the primary host of this ubiquitous mycoparasite. *Lophiotrema* sp. and *Antealophiotrema* sp. are interesting [68] but unexplored endophytic fungi (Figure 7). These species show morphological characteristics similar to those of conspecific fungal taxa [68,69,70], the fungicolous or mycoparasitic functions of which merit confirmation. Similarly, *Preussia* sp., such as endophytic *P. minima, P. africana,* and *P.* sp., may be important for biocontrol of plant pathogens [71] and/or insect larvae [72], as these modify chemical substances in plant tissues [73,74]. These species remained bioactive even under dry conditions [75], such as those prevailing during summer in Montenegro. Basidiomycetous *Sistotrema* sp., which is reportedly endophytic [76], may rather be considered a facultative fungicolous saprophyte, since it predominates in stage III trees, leading to increased wood decay. According to Bartnik [77], the presence of *S. brinkmannii* in wood during its saprotrophic phase is crucial for xylophagous insects, as this modifies the chemical composition, moisture content, and structure of wood, which, in turn, govern the survival and growth rate of larvae. This screening study for enhancing biocontrol potential against BTC is an immediate contribution to the pioneering studies of Bubák F. [78,79,80,81] and Japp O. [82] and Mijusković and Vucinić [83] on mycoparasitic mycoflora in Montenegro.

## 5. Conclusions

Modern biocontrol programs require more refined monitoring methods that enable the selection of promising fungicolous fungi, in order to better target and destroy stromata, thereby inhibiting the reproductive capacity of BTC pathogenic hosts. To date, the rDNA diversity profiles of fungicolous species have not been studied in relation to *Biscogniauxia* spp. The current study revealed that south European xylariaceous populations contained *B. destructiva* sp. Nov., an extremely destructive Tarcrust disease on *Fagus*. It appears that hybridization and introgression of *B. nummularia* and *B. anceps* might be main mechanisms to drive evolution and emergence of this new-Tarcrust pathogenic-fungus. Further, this study reveals a plethora of endophytic, mycoparasitic and/or hyperparasitic, fungicolous parasites that are possibly implicated in the natural biocontrol of Tarcrust across all stages of stroma development in BTC. The close relationship between some endophytic fungicolous taxa and mosses, lichens, and insects, which inhabit beech tree bark, merits further investigation. Hence, these fungicolous members can be considered not only as potential biocontrol candidates, but also as bioindicators of the complex, trophic relationship that exists between eukaryotic organisms forming different guilds in diseased beech bark. The study aims to shed light on potential natural biocontrol of the *Biscogniauxia* sp. nov.-Tarcrust disease that decimated *F. sylvatica* trees, leading to a decline in the ancient forest ecosystems of Montenegro.

## Figures and Tables

**Table 1:** Destructive fungicolous species that exhibit mycoparasitism on Xylariales Nannf. hosts (list updated from Sun et al. [13]).

Xylariaceous Fungus	Mycoparasite/Fungicolous Fungus	Origin [Ref]
*Xylariaceae* Tul. & C. Tul. (*Hypoxylon*, *Daldinia*, *Rosellinia*, and Xylaria)	*Acanthostigmella brevispina* M.E. Barr & Rogerson	USA, UK [13]
	*Cladobotryum campanisporum* G.R.W. Arnold	Cuba [4,14]
	*Hypocrea xylariicola* Henn	Brazil [5,15]
	*Muroia nipponica* I. Hino & Katum.	Japan [16]
	*Cytoplea parasitica* Petr., Feddes Repert	Pakistan [13]
	*Tubeufia brevispina* (M.E. Barr & Rogerson) J.L. Crane	USA [3]
	*Tubeufia cerea* (Berk. & M.A. Curtis) Höhn.	China [13], Poland [17]
	*Phragmogibbera xylariicola* Samuels & Rogerson	Venezuela [13]
	*Capronia moravica* (Petr.) E. Müll., Petrini, P.J. Fisher, Samuels & Rossman	USA [13]
	*Capronia parasitica* (Ellis & Everh E. Müll., Petrini, P.J. Fisher, Samuels & Rossman	USA [13]
	*Calcarisporium xylariicola* Jing Z.	Italy [3]
	*Neobarya xylariicola* Cand., J.D.	USA [3]
	*Polydesmia pruinosa* (Berk. & Broome) Boud.	France [18]
	*Hyphozyma lignicola* L.J. Hutchison	Canada [3]
	*Orbilia inflatula* (P. Karst.) P. Karst.	Germany [19]
	*Hydropisphaera hypoxantha* (Penz. & Sacc.) Rossman & Samuels	China [3]
	*Atkinsonella hypoxylon* (Peck) Diehl	USA [3]
	*Neobarya lutea* Samuels & Lodge	Puerto Rico [3]
	*Neobarya xylariicola* Cand., J.D. Rogers & Samuels	USA, France [3]
	*Hypocreopsis xylariicola* Samuels	Europe [3]
	*Trichoderma hypoxylon* Jing Z.	Guyana [13]
	*Trichoderma stilbohypoxyli* Samuels & Schroers	Thailand [13]
	*Cosmospora arxii* (W. Gams) Gräfenhan & Schroers	Puerto Rico [13]
	*Cosmospora vilior* (Starbäck) Rossman & Samuels	Canada [20]
	*Cosmospora episphaeria* (Tode) Rossman & Samuels *Nectria geastroides* Samuels	Germany [3]
	*Nectria viliuscula* Samuels, Yoshim. Doi & Rogerson	Worldwide [21]
	*Phaeoacremonium* sp. W. Gams, Crous et M. J. Wingf.	Amazonas [13]
	*Chlorostroma cyaninum* Læssøe, Srikit. & J. Fourn.	Indonesia [3]
	*Chlorostroma subcubisporum* A.N. Mill., Lar. N.	USA [3]
	Vassiljeva & J.D. Rogers	Thailand [3]
	*Acrostaphylus hypoxyli* G. Arnaud	USA [3]
	*Refractohilum mycophilum* Castañeda, W.B. Kendr. & Guarro	France [3]
	*Xenasma aculeatum* C.E. Gómez	Cuba [3]
	*Tremella flava* Chee J. Chen	Argentina [3]
	*Tremella resupinata* Chee J. Chen	China [13]
	*Tremella menglunensis* Y.B. Peng	China [13]
	*Mycogloea nipponica* Bandoni	China [13]
	*Immotthia hypoxylon* (Ellis & Everh.) M.E. Barr.	Japan [3], Poland [17]
*Diatrypaceae* Nitschke (*Diatrype*, *Diatrypetala*, *Eutype* and *Eutypella*)	*Capronia nigerrima* (R.R. Bloxam) M.E. Barr	UK [13]
	*Deltosperma infundibuliforme* W.Y. Zhuang	Trinidad-Tobago [13]
	*Chaetosphaeria phaeostroma* (Durieu & Mont.) Fuckel	Europe [18]
	*Chaetosphaerella fusca* (Fuckel) E. Müll. & C. Booth	Europe [18]
	*Chaetosphaerella phaeostroma* (Durieu & Mont.) E. Müll. & C. Booth	Europe [8]
	*Nectria pseudepisphaeria* Samuels	USA [13]
	*Nectria triqua* Samuels	French Guiana [3]
	*Ophiostoma grande* Samuels & E. Müll.	Brazil [3]
	*Diplococcium heterosporum* L. Zeller & Tóth	Hungary [13]
	*Endophragmiella eboracensis* B. Sutton	Canada [13]
	*Annellodochium ramulisporum* Deighton	Sierra Leone [13]
	*Tomentella badiofusca* f. *diatrypicola* Svrček,	Czechoslovakia [13]
	*Sirobasidium sandwicense* Gilb. & Adask.	USA [13]
	*Tremella episphaerica* Rick	China [13]
	*Tremella nivalis* Chee J. Chen	China [13]
	*Achroomyces henricii* P. Roberts	USA [3]
	*Chloridium clavaeformae* (Pr.) Gams et Hol.-Jech.	Poland [17]
Other	*Ampelomyces* sp. Ces.	Canada [5]
	*Aureobasidium pullulans* (de Bary) G. Arnaud	
	*Gliocladium* sp. Corda	
	*Cladosporium herbarum* (Pers.: Fr.) Link	
	*Cladosporum cladosporioides* (Pers.) Link	
	*Epicoccum nigrum* Link	
	*Leptographium microsporum* R.W. Davidson	
	*Paecilomyces farinosus* (Holm.) Brown & SM.	
	*Trichoderma harzianum* Rifai	
	*Trichoderma viride* Pers.: Fr.	
	*Verticillium lecanii* (Zimm.) Viegas	
	*Scolicosporium* sp. Lib. ex Roum.	

**Table 2:** Distinctive taxonomic characteristics between sister ***Biscogniauxia*** species.

*Biscogniauxia* Taxon	*Anceps*	*Nummularia*	*Destructiva*
**Stroma shape**	applanate, irregularly orbicular 5–25 mm diam	applanate, discoid 5–20 mm diam	applanate, discoid 5–30 mm diam
**Stroma colour**	dull black, carbonaceous	black, carbonaceous	black, carbonaceous
**Ostioles**	umbilicate	umbilicate or papillate	umbilicate or papillate
**Ascospore septation**	two-celled	one-celled	one or two-celled
**Ascospore colour**	hyaline (abundant) and brown or blackish brown	hyaline (rare) and brown	Non-hyaline, brown and blackish brown
**Ascospore shape**	Sub-globose, broadly ellipsoid to deltoid (larger-dark cell) with cuneate extrusion (inferior, smaller-less colored membranous cell)	narrowly ellipsoid, ellipsoid to fusiform, without appendage	ovoid, ellipsoid to deltoid (larger-dark cell) and heap shaped extrusion (inferior, smaller-less colored membranous cell)
**Ascospore size**	13–16 (18) × 7–8 (9) µm	11.5–13.5(16) × 8.–9.5 (11) µm	12–16 (18) × 8–11 (12) µm
**Appendage**	Straight, stick-like appendage with narrow-hyaline apex, 2–3 µm long	no appendage	Curved, thread-like appendage with a broadened-dark apex, 3–5 µm long.
